# Supplementary material for: Molecular interplay between HURP and Kif18A in mitotic spindle regulation
Source: Res Sq. 2024 May 29:rs.3.rs-4249615. Preprint. [Version 1] doi: 10.21203/rs.3.rs-4249615/v1 (PMC11160874; doi:10.21203/rs.3.rs-4249615/v1)
Supplement: 1 [file NIHPPrs4249615V1-supplement-1.pdf]

Supplementary Figures and Tables

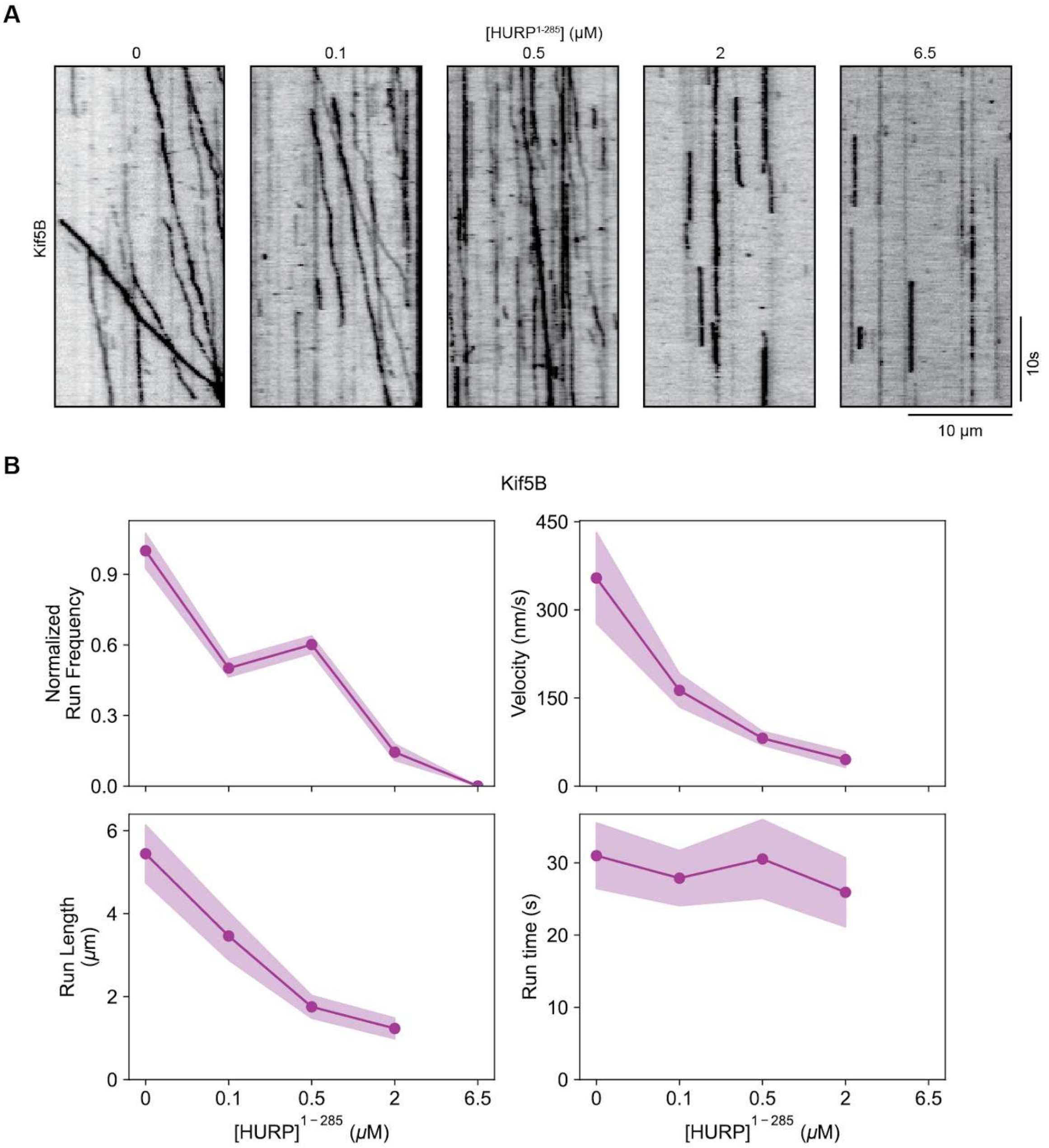

Supplementary Figure 1. HURP fails to activate Kif5B motility. A. Representative kymographs showing the motility of full-length Kif5B in the presence of increasing HURP$^{1\text{-}285}$. **B**. Normalized run frequency (n = 10 kymographs for each condition), velocity, run length and run time (from left to right, n = 25 motors for each condition) of Kif5B for different HURP$^{1\text{-}285}$ concentrations. The line and shadows represent the mean and S.E., respectively.

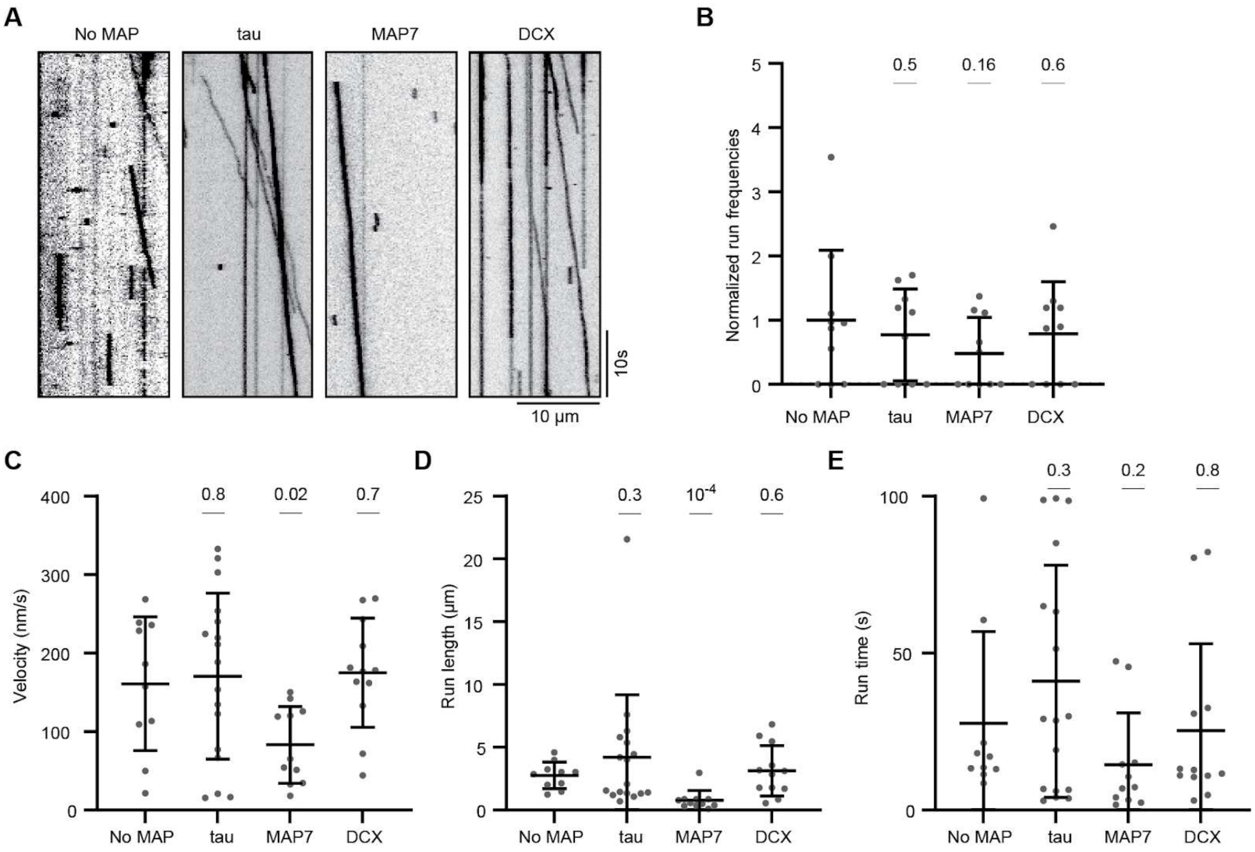

Supplementary Figure 2. Tau, MAP7 and DCX fail to activate Kif18A motility. A. Representative kymographs showing motility of Kif18A in the presence of tau, MAP7 and DCX. **B - E.** Normalized run frequency (**B**) , velocity (**C**), run length (**D**) and run time (**E**) of Kif18A in the presence of tau, MAP7 or DCX (For **B**, n = 10 kymographs for each condition; for **C** - **E**, n = 10, 17, 11, 12 motors). The center line and whiskers represent the mean and S.D., respectively. P values are calculated from a two-tailed t test.

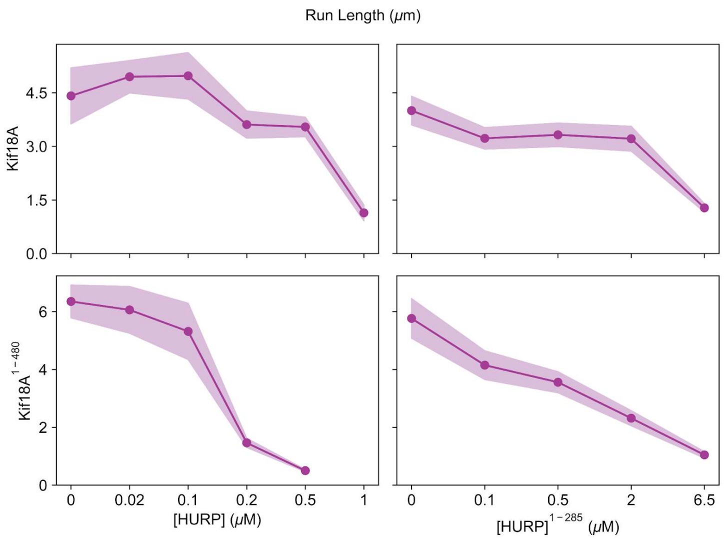

Supplementary Figure 3. High concentrations of HURP restrict the run length of Kif18A. A. Run length of Kif18A with titrated HURP (upper left, n = 32, 50, 33, 48, 50, 40 motors, respectively), Kif18A with titrated HURP$^{1\text{-}285}$ (upper right, n = 51, 44, 54, 52, 52 motors, respectively), Kif18A$^{1\text{-}480}$ with titrated HURP (lower left, n = 25 motor for each data point) and Kif18A$^{1\text{-}480}$ with titrated HURP$^{1\text{-}285}$ (lower right, n = 52, 51, 52, 52, 52 motors, respectively). The line and shadows represent the mean and S.E., respectively.

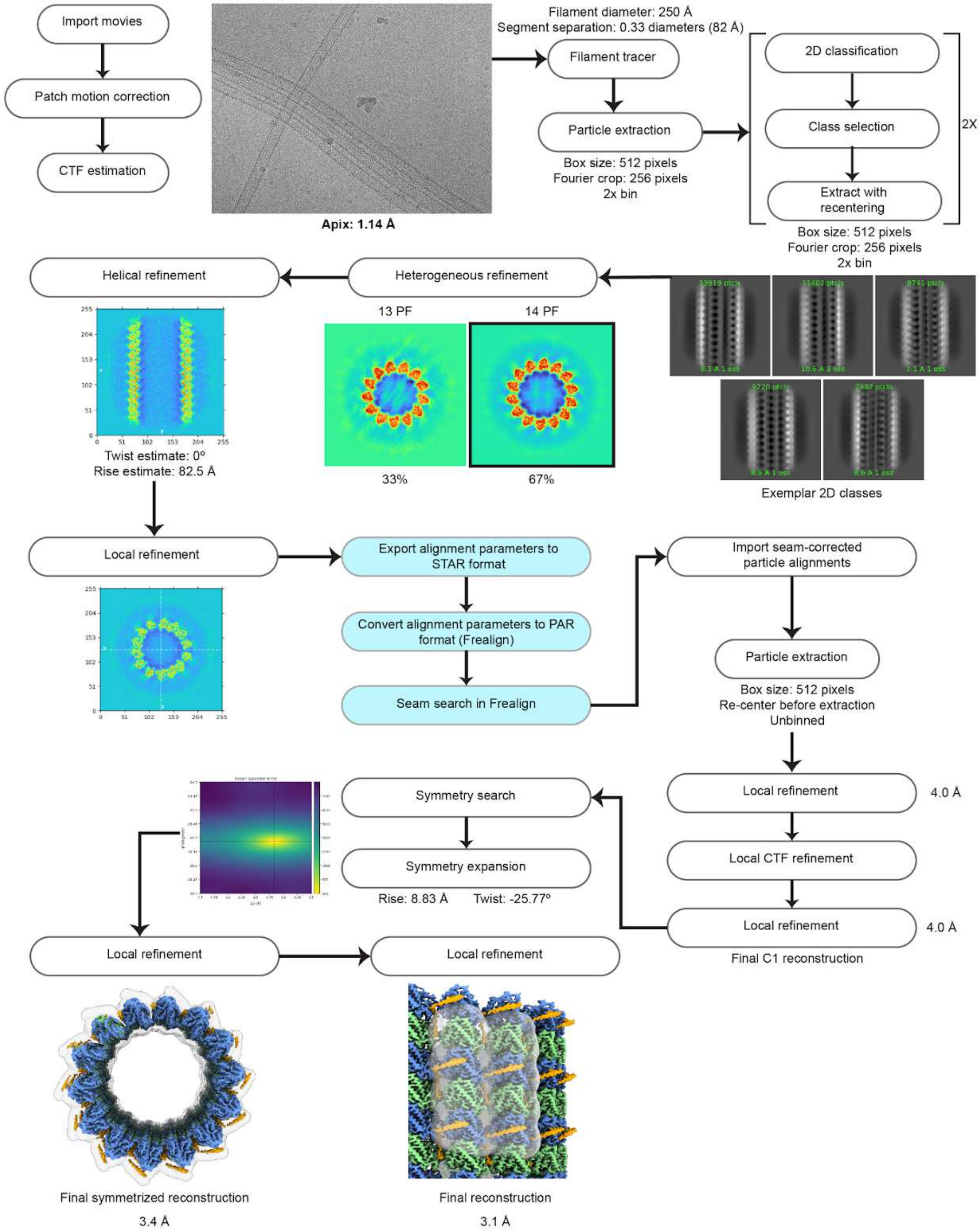

Supplementary Figure 4. Cryo-EM data processing for the microtubule-HURP dataset.

Processing pipeline applied to the HURP-bound microtubule dataset. Unless specified otherwise, all

steps were performed in CryoSparc. Boxes in light blue include steps implemented outside of the CryoSparc software package. Masks are shown with transparent surfaces.

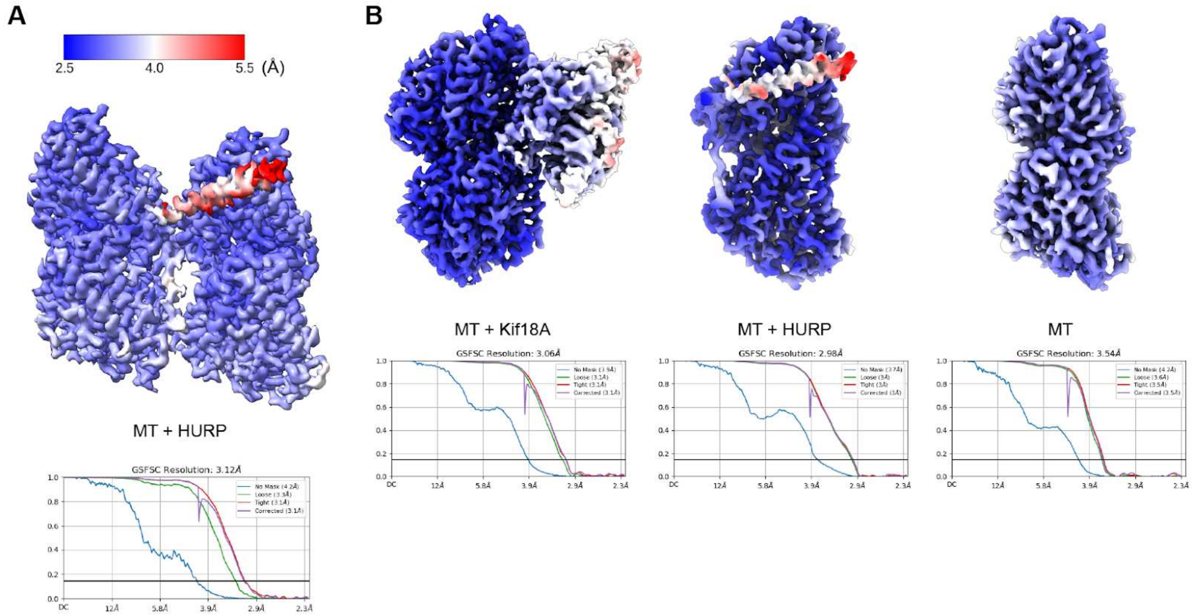

Supplementary Figure 5. Local resolution maps and FSC curves. A. Local resolution map (top) and Fourier Shell Correlation (FSC) plots (bottom) for microtubule-bound HURP. **B.** Local resolution maps (top) and Fourier Shell Correlation (FSC) plots (bottom) for the three classes produced after 3D classification of the microtubule-HURP-Kif18A$^{1-373}$ dataset.

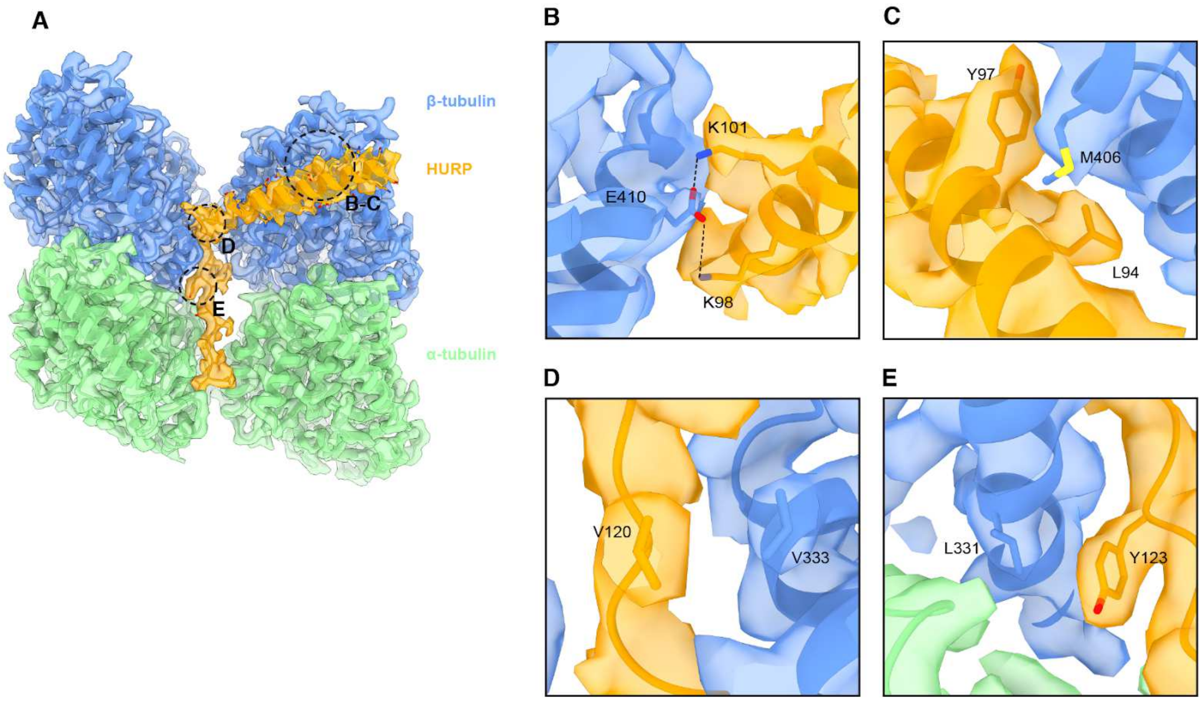

Supplementary Figure 6. Detailed interactions between HURP and tubulin. A. Final microtubule-HURP cryo-EM map. A single HURP molecule is shown for clarity. The refined model is overlaid on the map. Dashed circles indicate the regions shown in panels B-E. **B-E.** Additional interactions between HURP and tubulin.

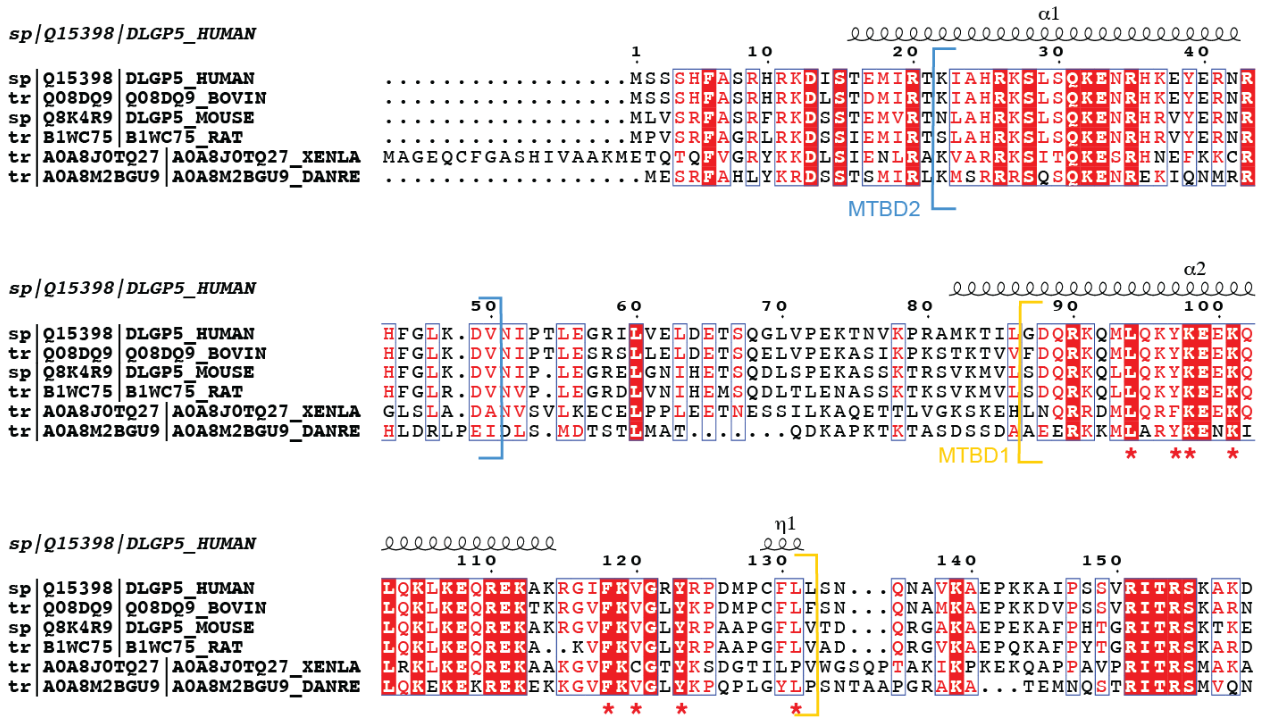

Supplementary Figure 7. HURP residues that interact with tubulin are conserved across species. Sequence alignment and conservation analysis for HURP from different species. The human version was set as a reference and residues that are seen to interact with tubulin in our cryo-EM structure are marked with red asterisks. ESPript3[70] was used to generate the figure, and secondary structure was annotated based on the AlphaFold[71] prediction for human HURP (Uniprot Q15398). Regions corresponding to MTBD2 (blue) and the structurally resolved part of MTBD1 (yellow) are indicated.

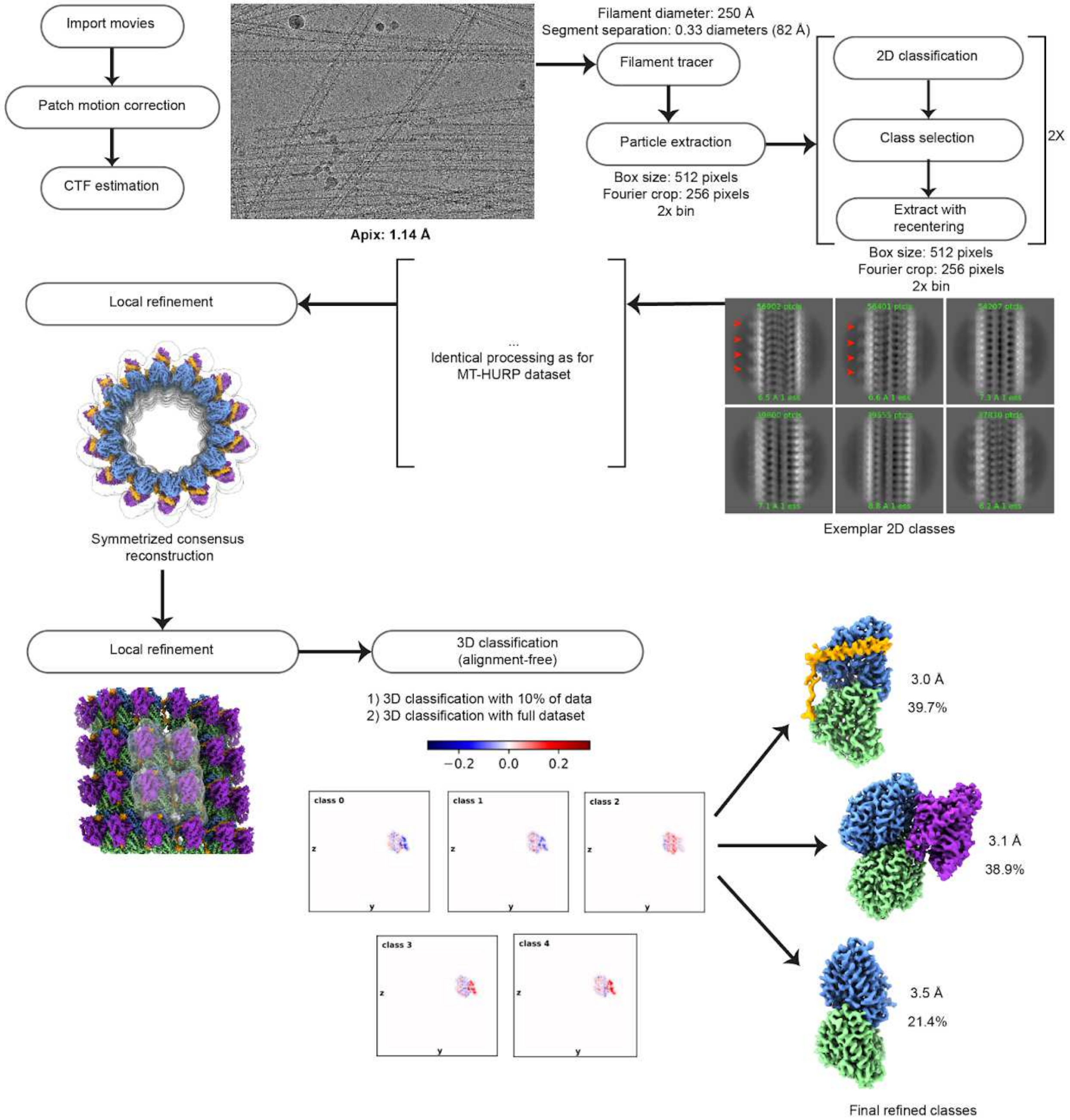

Supplementary Figure 8. Cryo-EM data processing for the microtubule-HURP-Kif18A dataset. Processing pipeline applied to the microtubule+HURP+Kif18A$^{1\text{-}373}$ dataset. Unless specified otherwise, all steps were performed in CryoSparc. Red arrowheads point to Kif18A density in the 2D class averages. In the 3D classification step, the blue-red key represents density variation in

each class with respect to the input consensus reconstruction, with regions in blue and red containing significantly less or more density than the consensus, respectively. Masks are shown with transparent surfaces.

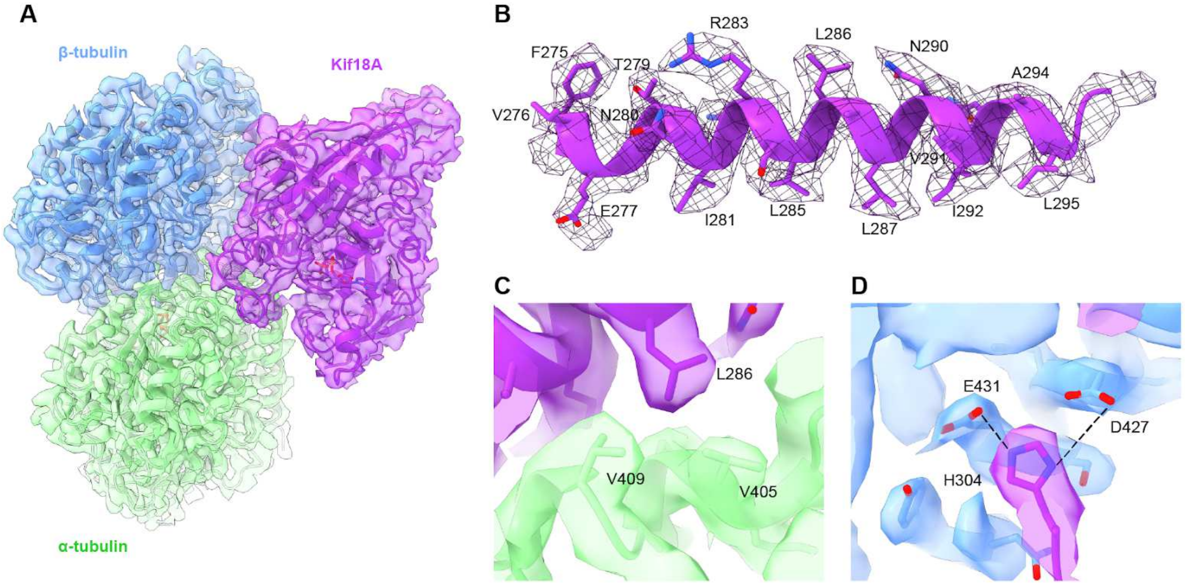

Supplementary Figure 9. Map quality and detailed interactions for the Kif18A-tubulin class. **A.** Surface representation of the cryo-EM density corresponding to the microtubule+Kif18A class, with a refined tubulin+Kif18A model fitted inside the map. α-tubulin, *β*-tubulin and Kif18A are represented in green, blue and purple, respectively. **B.** Isolated density in mesh representation for Kif18A's α4 helix, with the corresponding segment of the real-space refined atomic model of Kif18A. **C-D.** Selected detailed interactions between Kif18A and tubulin.

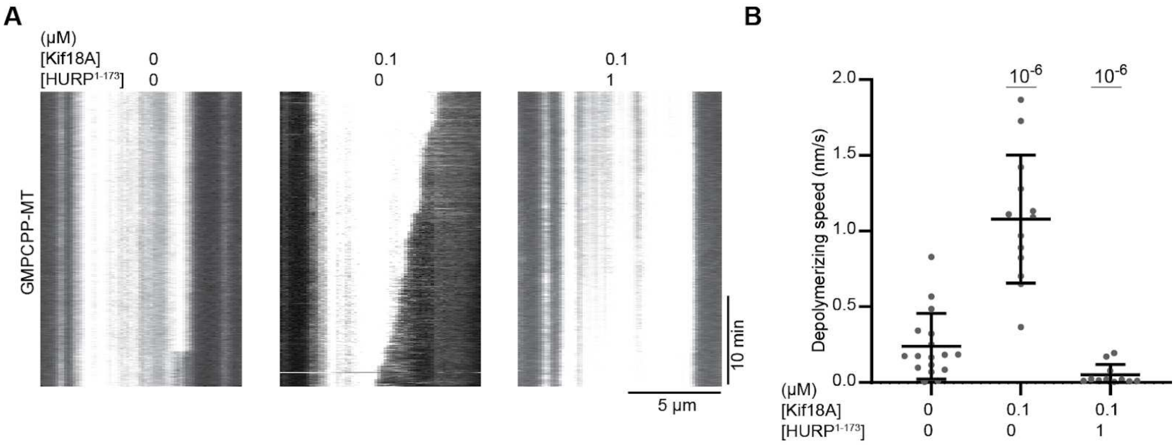

Supplementary Figure 10. Kif18A can depolymerize GMPCPP microtubules. A. Representative kymographs of GMPCPP-microtubule depolymerization with 0.1 μM Kif18A or 0.1 μM Kif18A + 1 μM HURP$^{1-173}$. **B**. Microtubule depolymerization speed with 0.1 μM Kif18A or 0.1 μM Kif18A + 1 μM HURP$^{1-173}$. (From left to right, n = 17, 13 and 11 kymographs). The center line and whiskers represent the mean and S.D., respectively. P values are calculated from a two-tailed t test.

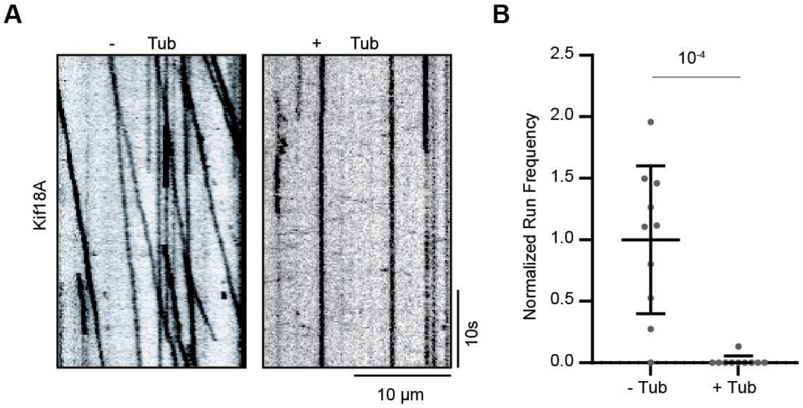

Supplementary Figure 11. Kif18A motility is affected by the presence of free tubulin. A. Representative kymographs showing the motility of full-length Kif18A with or without 2 mg/mL free tubulin. **B**. Normalized run frequency of full-length Kif18A with or without 2 mg/mL free tubulin (n = 10 kymographs for each condition). The center line and whiskers represent the mean and S.D., respectively. P values are calculated from a two-tailed t test.

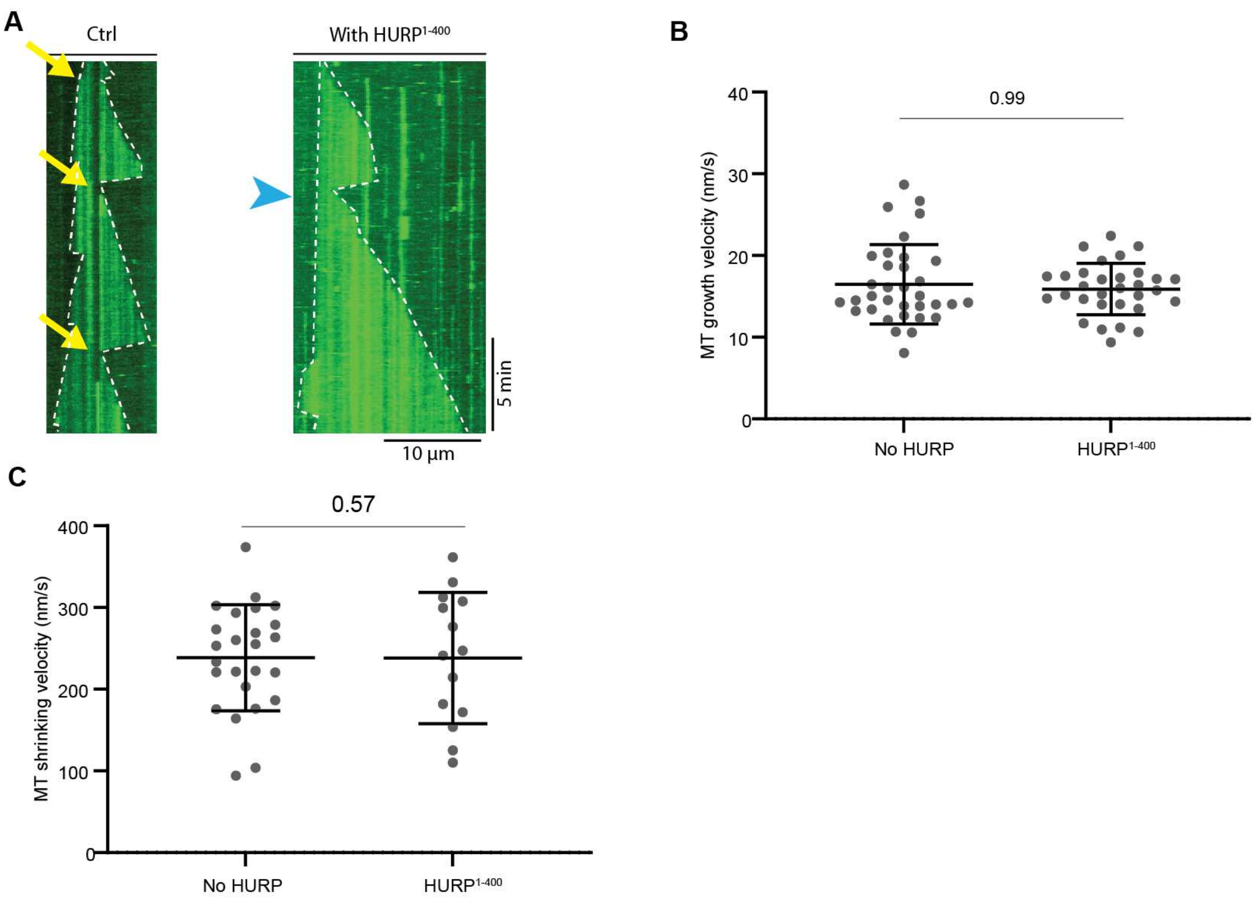

Supplementary Figure 12. Effect of HURP$^{1\text{-}400}$ on dynamic microtubules. A. Kymographs of dynamic microtubules with or without HURP$^{1\text{-}400}$. Yellow arrows represent catastrophe events and blue arrowheads represent rescue events. **B.** Microtubule plus-end growth velocities with or without HURP$^{1\text{-}400}$ (from left to right, n = 34, 31 microtubule growth periods). **C.** Microtubule plus-end shrinking velocities with or without HURP$^{1\text{-}400}$ (from left to right, n = 25, 14 microtubule shrinking periods). In **B**-**C**, the center line and whiskers represent the mean and S.D., respectively. P values shown above the data points are calculated from a two-tailed t test.

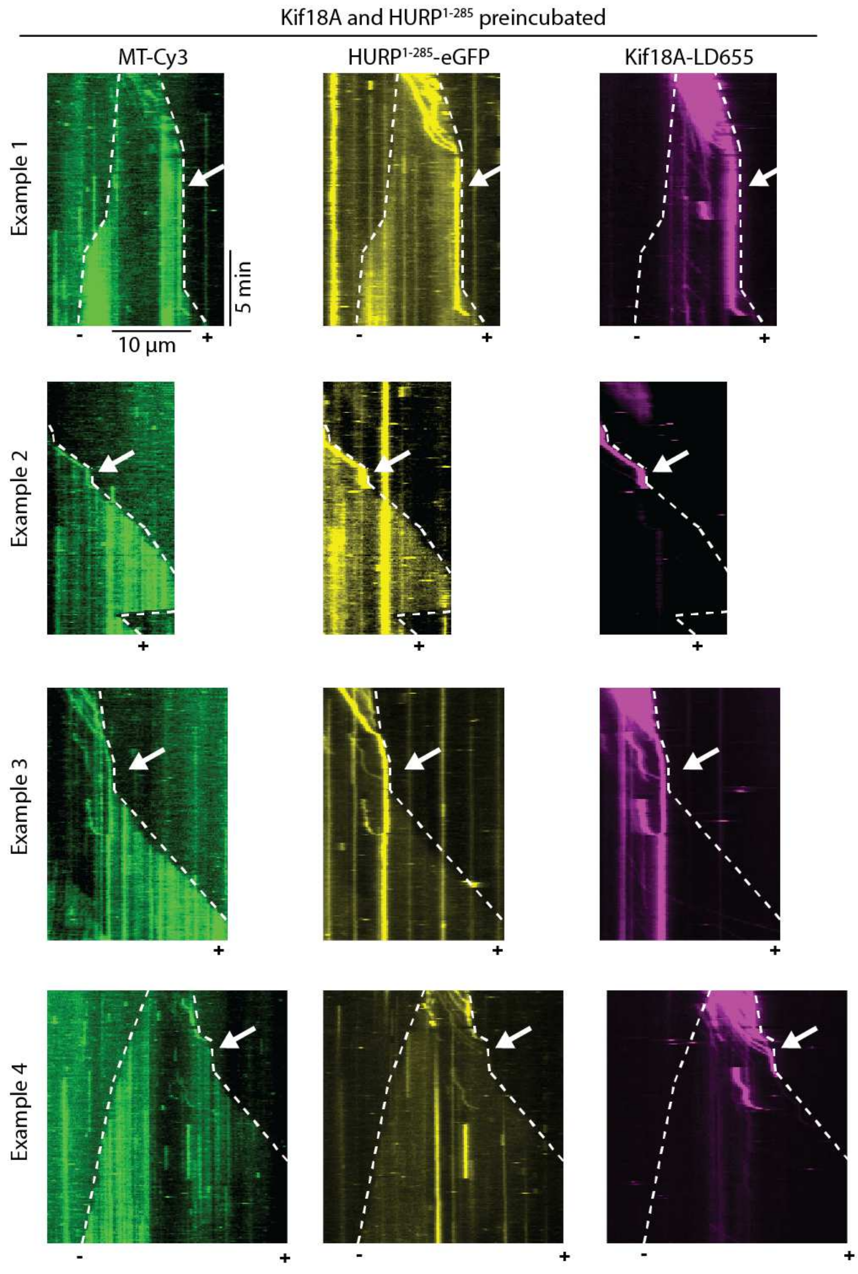

Supplementary Figure 13. Kif18A and HURP jointly control microtubule length. Kymographs of Kif18A and $HURP^{1\text{-}285}$ collectively maintaining a constant microtubule length (shown by white arrow). White dashed lines show the track of microtubule ends.

Name	MT + HURP	MT + HURP + Kif18A (HURP class)	MT + HURP + Kif18A (Kif18A class)	MT + HURP + Kif18A (Tubulin class)
EMDB ID	XXX	XXX	XXX	XXX
Microscope	Arctica	Arctica	Arctica	Arctica
Voltage (kV)	200	200	200	200
Camera	K3	K3	K3	K3
Defocus range (μm)	0.8-2	0.8-2	0.8-2	0.8-2
Automation software	SerialEM	SerialEM	SerialEM	SerialEM
Frames	50	50	50	50
Total dose (electrons/Å^2)	50	50	50	50
Pixel size (Å/pixel)	1.14	1.14	1.14	1.14
Number of micrographs	796	2611	2611	2611
Starting number of particles (pre-symmetry expansion)	99,992	1,151,884	1,151,884	1,151,884
Number of particles in final map (post-symmetry expansion)	353,980	2,405,078	2,349,581	1,296,716
Map sharpening B factor (Å)	-60	-100	-60	-80
Map sharpening methods	CryoSparc	CryoSparc	CryoSparc	CryoSparc
Symmetry	C1	C1	C1	C1
Overall resolution (Å)	3.1	3.0	3.1	3.5
Resolution range of map (min-75th percentile) (Å)	2.8-4.1	2.5-5.6	2.6-4.0	2.9-4.4

Metric	Value	
	MT + HURP	MT + Kif18A
Initial model used (PDB ID)	6DPV	5OCU
Refinement package	Phenix	Phenix

C-beta outliers (%)	0	0
Rotamer outliers (%)	0.34	0.1
All-atom Clash score	9.17	8.76
MolProbity score	1.94	2.01
Ramachandran plot (outliers/favored) (%)	0.64/93.00	0.44/90.39
Ligand	8	6
Protein residues	1724	1157
R.m.s.d. of bond lengths (Å)	0.003	0.004
R.m.s.d. of bond angles (°)	0.644	0.733
CC (mask)	0.84	0.81
CC (volume)	0.84	0.75

Table S1. Cryo-EM data collection parameters and model refinement statistics
